# A Chemically Defined Medium for Rabbit Embryo Cryopreservation

**DOI:** 10.1371/journal.pone.0071547

**Published:** 2013-08-20

**Authors:** Pierre Bruyère, Anne Baudot, Thierry Joly, Loris Commin, Elodie Pillet, Pierre Guérin, Gérard Louis, Anne Josson-Schramme, Samuel Buff

**Affiliations:** 1 UPSP 2011.03.10 (ICE), VetAgro Sup (Université de Lyon), Marcy l'étoile, France; 2 INSERM U698, Université Paris-Descartes (PRES Sorbonne Paris Cité), Paris, France; 3 Département des productions animales, ISARA-Lyon, Lyon, France; Universitat Pompeu Fabra, Spain

## Abstract

This study evaluates a new synthetic substitute (CRYO3, Ref. 5617, Stem Alpha, France) for animal-based products in rabbit embryo cryopreservation solutions. This evaluation was performed using two approaches: a thermodynamic approach using differential scanning calorimetry and a biological approach using rabbit embryo slow-freezing. During the experiment, foetal calf serum (FCS) was used as a reference. Because FCS varies widely by supplier, three different FCS were selected for the thermodynamic approach. The rabbit embryo slow-freezing solutions were made from Dulbecco's phosphate buffer saline containing 1.5 M Dimethyl Sulfoxide and 18% (v.v^−1^) of CRYO3 or 18% (v.v^−1^) of FCS. These solutions were evaluated using four characteristics: the end of melting temperature, the enthalpy of crystallisation (thermodynamic approach) and the embryo survival rates after culture and embryo transfer (biological approach). In the thermodynamic approach, the solutions containing one of the three different FCS had similar mean thermodynamic characteristics but had different variabilities in the overall data with aberrant values. The solution containing CRYO3 had similar thermodynamic properties when compared to those containing FCS. Moreover, no aberrant value was measured in the solution containing CRYO3. This solution appears to be more stable than the solutions containing a FCS. In the biological approach, the *in vitro* embryo survival rates obtained with the solution containing CRYO3 (73.7% and 81.3%) and with the solution containing a FCS (77.6% and 71.9%) were similar (*p* = 0.7). Nevertheless, during the *in vivo* evaluation, the implantation rate (21.8%) and the live-foetuses rate (18.8%) of the CRYO3 group were significantly higher than the implantation rate (7.1%, *p* = 0.0002) and the live-foetuses rate (5.3%, *p* = 0.0002) of the FCS group. The pregnancy rate was also higher in the CRYO3 group compared to the FCS group (81.3% and 43.8%, respectively, *p* = 0.066). We conclude that CRYO3 can be used as a chemically defined substitute for animal-based products in rabbit embryo cryopreservation solutions.

## Introduction

Embryo transport and transfer in domestic animals is widely used because it is considered to be safest, less expensive and more respectful to animal welfare than animal transport [1]. In 2010, more than 930,000 *in vivo*- or *in vitro*-produced bovine embryos were transferred worldwide, including more than 350,000 frozen/thawed embryos [2].

However, although embryo transfer is considered to be safe according to the recommendations of the International Embryo Transfer Society, sanitary risks still exist [1], [3], [4]. One of these concerns is the source of macromolecules added to the various media used for embryo transfer [5], including the cryopreservation solutions. In fact, the most frequently used sources for cryopreservation are animal-derived products, such as foetal calf serum (FCS) or bovine serum albumin (BSA), which may be contaminated by pathogens, particularly by viruses [4], [6], [7]. Among these viruses, Bovine Viral Diarrhoea virus is the most important cause of concern because of its large economic impact [8], its prevalence in FCS [9], its relative resistance to gamma irradiation [6] and cryopreservation procedures [10], and consequently the associated risk of disease transmission to recipients by an infected embryo [11]. Prion diseases can also probably be transmitted through blood-derived products [12]–[14], especially as it is difficult to inactivate the transmissible degenerative encephalopathy agents with the usual processing of animal-based products [15]. In addition to the sanitary risks, undefined compounds, especially peptides, may be bound to albumin and may induce variability in the composition of the FCS or BSA, causing deleterious effects on embryos [16].

Although using BSA lowers the sanitary risks [1], [17], the safest practice is to use substitutes that are not biologically derived [17], [18]. Sodium hyaluronate [5], [19] and vegetable peptones [20] have been successfully used as substitutes for animal products in embryo cryopreservation solutions. Acceptable but less consistent results have also been obtained with polyvinyl alcohol [21]–[25]. The use of polyvinylpyrrolidone [21], [26] or surfactants (VF 5 [27], Pluronic F68 [19]) has led to less conclusive results. Dextran [28] and ficoll [25], [28] have also been added to embryo cryopreservation solutions without any animal-derived macromolecular components to reduce the concentrations of cryoprotectants.

However, the use of substitutes may lead to variations in the physical properties of the cryopreservation solutions that could be deleterious for the embryos. Consequently, these variations need to be controlled. Differential Scanning Calorimetry (DSC) is an interesting physical analysis tool that has been used in cryobiology to characterise cryopreservation solutions [29], [30], to optimise cryopreservation protocols [31]–[34], and to better understand ice formation in cells, embryos and other living organisms [35]–[38]. In fact, DSC allows the dynamic evaluation of thermal properties of cryopreservation solutions, particularly during crystallisation and melting. For slow-freezing solutions, the thermodynamic characteristics, such as the phase transition temperature and the quantity of ice crystallised and melted, can thus be measured.

In a preliminary study, Joly obtained promising results in rabbit (*Oryctolagus cuniculus*) embryo cryopreservation with a chemically defined substitute that contained no animal products (CRYO3, Ref. 5617, Stem Alpha, France) [39]. The aim of this study is to more thoroughly evaluate this potential substitute with two consecutive approaches. First, DSC was used to compare selected thermodynamic characteristics of cryopreservation solutions containing the potential substitute (CRYO3) with those of standard cryopreservation solutions containing FCS. Second, the substitute was evaluated in a biological setting by quantifying the viability after *(i) in vitro* culture and *(ii)* embryo transfer of rabbit embryos which were previously frozen in a solution containing the substitute or a FCS-based solution.

## Results

### Thermodynamic approach

#### Thermodynamic characteristics of the cryopreservation solutions containing FCS

As shown in Table 1, the mean values of the end of melting temperature (*T*_m_) and the enthalpy of crystallisation (Δ*H*) were similar for the cryopreservation solutions containing one of the three FCSs (FCS solutions).

**Table 1:** Thermodynamic characteristics of the cryopreservation solutions (Mean ± standard deviation).

	*T*_m_ (°C)	Δ*H* (J.g^−^1)
Solution without any MC^a^ (n = 9, control solution)	−3.33±0.27	185.06±2.62
Solution containing FCS^b^ 1 (n = 9)	−3.01±0.38	180.49±2.54
Solution containing FCS 2 (n = 9)	−3.15±0.28	180.82±3.61
Solution containing FCS 3 (n = 9)	−3.12±0.38	178.36±7.88
Pooled solutions containing FCS (n = 27)	−3.10±0.34	179.89±5.13
Solution containing CRYO3 (n = 9)	−2.76±0.36	183.39±3.58

a : macromolecular component.

b : foetal calf serum.

**Figure 1:**
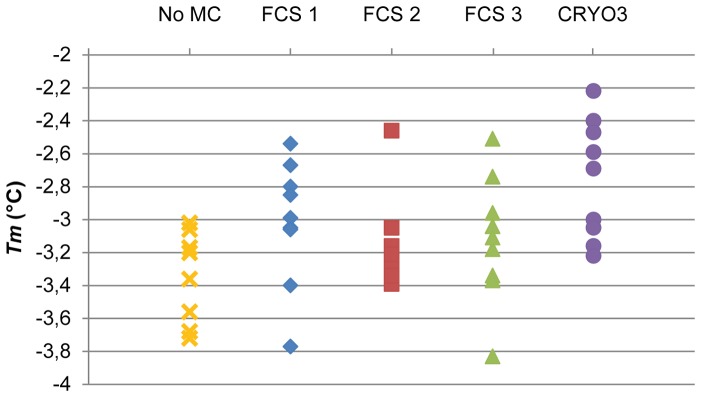
Diagram of the *T*_m_ values for the cryopreservation solutions. MC: macromolecular component; FCS: foetal calf serum.

**Figure 2:**
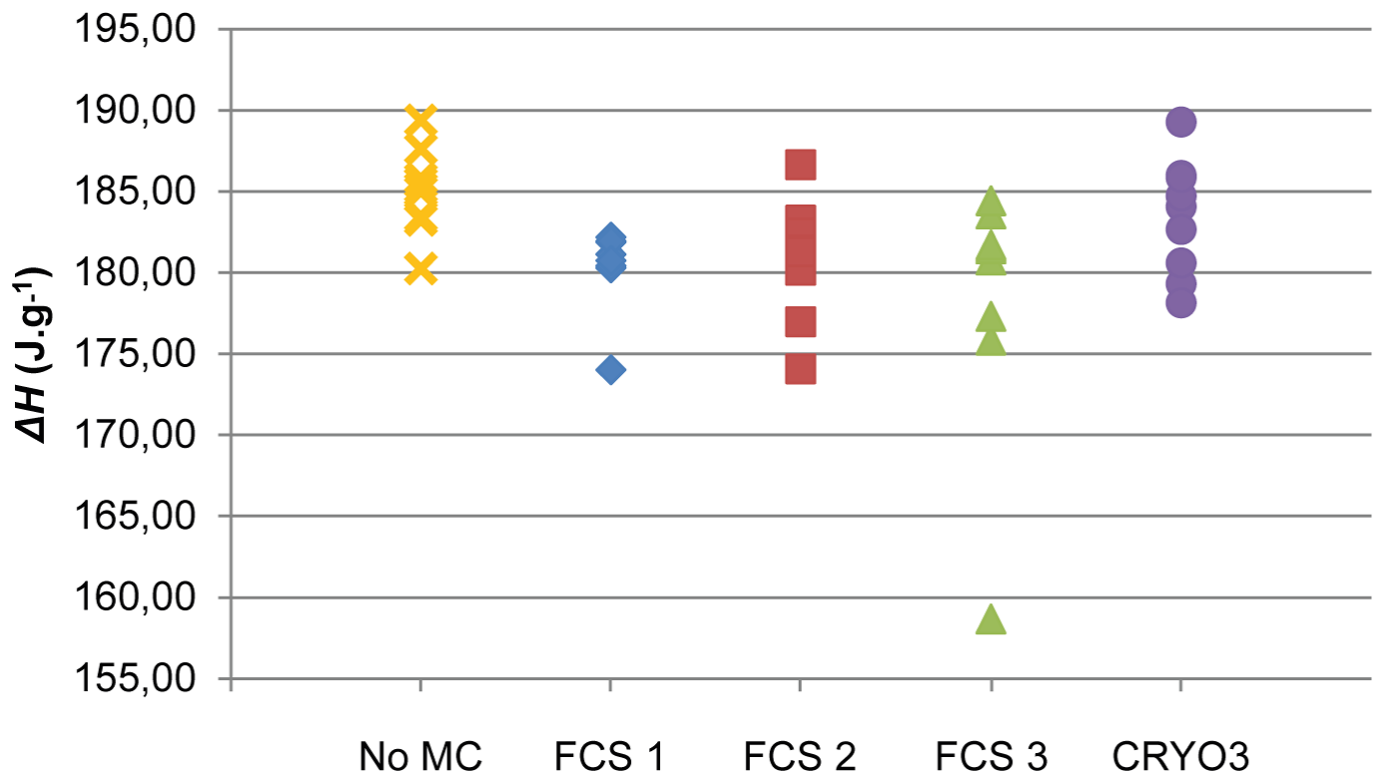
Diagram of the Δ*H* values for the cryopreservation solutions. MC: macromolecular component; FCS: foetal calf serum.

Conversely, the distributions of the *T*_m_ and Δ*H* values were relatively different among the different FCS solutions. The *T*_m_ had the lowest variations in the solution containing FCS 2 (Fig. 1). The *T*_m_ of the solutions containing FCS 1 and FCS 3 had similar distributions and seemed to produce greater variations. The Δ*H* of the solutions containing FCS 1 or FCS 3 seemed to be the most stable (Fig. 2). Nevertheless, an aberrant value was measured with the solution containing FCS 3. More variations were observed with the solutions containing FCS 2.

The various FCSs had diverse effects on the thermodynamic characteristics of the cryopreservation solutions. FCS seemed to slightly increase the mean values of the *T*_m_ obtained with cryopreservation solutions. However, FCS 2 and FCS 3 showed less influence than FCS 1 (Table 1). All FCSs slightly decreased the mean values of Δ*H* obtained with cryopreservation solutions.

The different FCSs variably influenced the distributions of *T*_m_ and Δ*H*. Thus, although FCSs seemed to slightly increase the variability of the *T*_m_ results (Fig. 1), the solution containing FCS 2 had variations that were relatively close to those obtained with the control solution whereas the variations obtained with solutions containing FCS 1 or FCS 3 were higher than those obtained with the control solution. In the same way, excluding the aberrant value, the variations in Δ*H* for the solutions containing FCS 1 or FCS 3 were similar to those obtained with the control solution (Fig. 2), while the solution containing FCS 2 had variations that were slightly higher than those obtained with the control solution.

**Figure 3:**
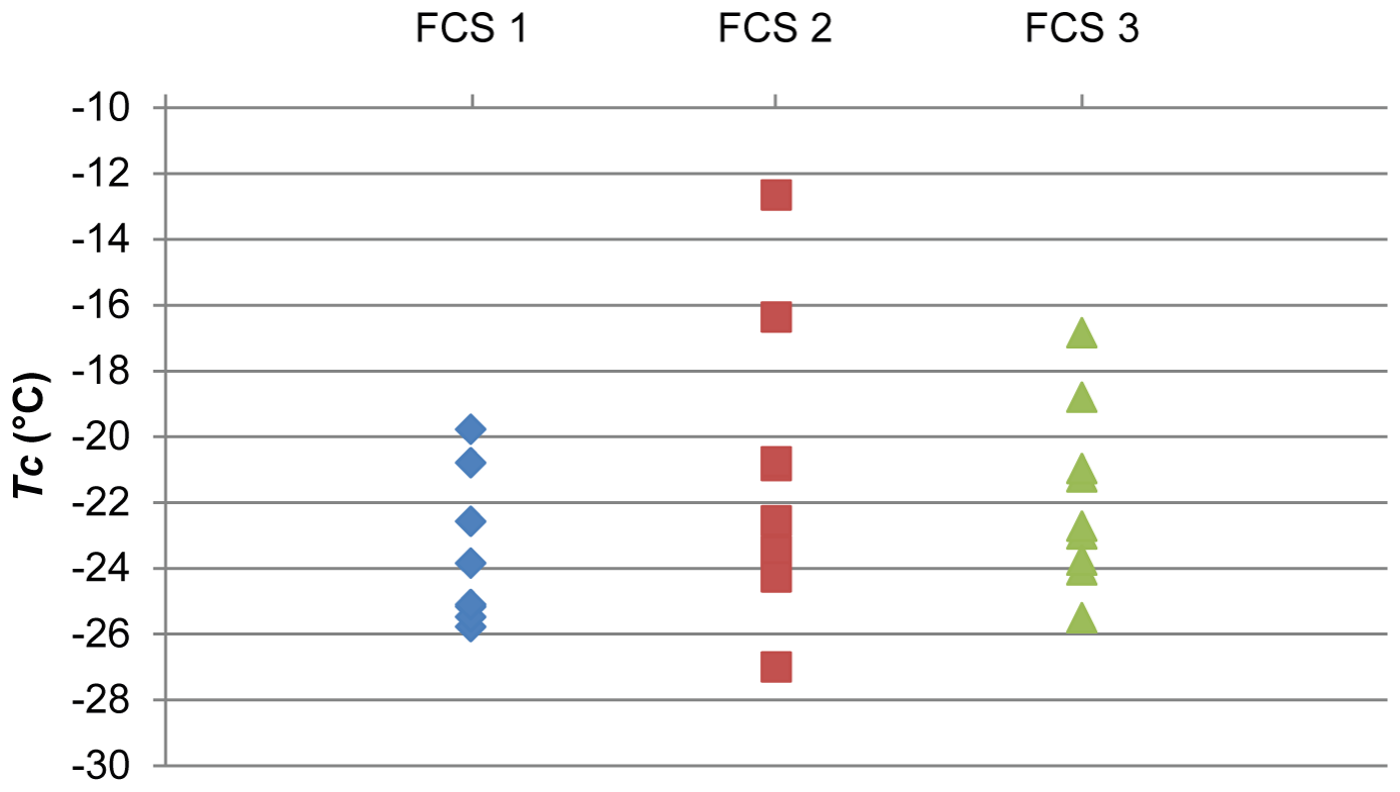
Diagram of the *T*_c_ values obtained with the cryopreservation solutions containing FCS. FCS: foetal calf serum.

Two of the tested FCSs could be considered thermodynamically stable under our experimental DSC conditions (weight of samples, kind of pans, range of temperature, cooling and warming rates): FCS 2 for *T*_m_ and FCS 1 for Δ*H*. However, the solution containing FCS 1 was also the most thermodynamically stable for the crystallisation temperature during slow cooling (*T*_c_) in our experimental conditions (Fig. 3). Consequently, FCS 1 was chosen for comparison in the biological approach.

#### Thermodynamic characteristics of the cryopreservation solution containing CRYO3

The mean values of *T*_m_ and Δ*H* were similar among all of the cryopreservation solutions containing a single type of FCS. Consequently, the mean properties of pooled FCS solutions were compared with those of the solution containing CRYO3 (CRYO3 solution).

The thermodynamic characteristics of the CRYO3 solution were relatively close to those obtained with FCS solutions. Concerning *T*_m_, the different values obtained with the CRYO3 solution ranged in the same order of magnitude as those obtained with FCS solutions according to the experimental precision (Fig. 1). Moreover, the variations obtained with the CRYO3 solution were similar to those obtained with the solution containing the most thermodynamically stable FCS (FCS 2) and were slightly lower than those obtained with solutions containing one of the other two FCSs. Regarding the mean values of *T*_m_, there was a slight difference between that obtained with the CRYO3 solution and that obtained with pooled FCS solutions (Table 1).

The values of Δ*H* for the CRYO3 solution were also close to those obtained with FCS solutions. The variations in the CRYO3 solution were *(i)* slightly higher than those obtained with the solution containing the most thermodynamically stable FCS (FCS 1), *(ii)* similar to those obtained with the solution containing FCS 2 and *(iii)* lower than those obtained with the solution containing FCS 3. The mean value of Δ*H* for the CRYO3 solution was close to that for pooled FCS solutions (Table 1).

### BiologSPAINal approach

The results of the *in vitro* and *in vivo* viability assays of the frozen/thawed embryos are shown in Table 2. Five hundred and fifty five embryos were cultured or transferred during the study (*n* = 277 in the FCS group (FCS 1); *n* = 278 in the CRYO3 group).

**Table 2:** Embryo viability after *in vitro* and *in vivo* evaluation.

	*In vitro* evaluation^a^		*In vivo* evaluation		
	First evaluation	Second evaluation	Pregnancy rate^b^	Implantation rate^c^	Live-foetuses rate^d^
FCS group	77.6% (59/76)	71.9% (23/32)	43.8% (7/16)	7.1% (12/169)	5.3% (9/169)
CRYO3 group	73.7% (56/76)	81.3% (26/32)	81.3% (13/16)	21.8% (37/170)	18.8% (32/170)
Significance	*p* = 0.7	*p* = 0.55	*p* = 0.066	*p* = 0.0002	*p* = 0.0002

a : development in blastocysts or expanded blastocysts after 48 h of *in vitro* culture.

b : number of doe containing at least one foetus/total recipient does.

c : number of implantation sites/total embryos transferred into recipient.

d : number of living foetuses/total embryos transferred into recipient.

#### In vitro evaluation of embryo viability

Two in vitro evaluations were performed. The second in vitro evaluation was performed at the same time as the in vivo evaluation.

During the first *in vitro* evaluation, only three out of 76 embryos of each group were morphologically damaged (mucin coat damaged or with inclusions). After *in vitro* culture, the embryo survival rate for the CRYO3 group was close to that observed for the FCS group (73.7% and 77.6%, respectively). No significant difference was observed between the two groups (*p* = 0.7).

During the second *in vitro* evaluation, no embryo was morphologically damaged in each group of 32 embryos. The embryo survival rates were not significantly different between the CRYO3 group and the FCS group (81.3 and 71.9%, respectively, *p* = 0.55) and were not significantly different from those obtained during the first *in vitro* evaluation of viability (*p* = 0.47 for the CRYO3 group and *p* = 0.62 for the FCS group).

#### In vivo evaluation of embryo viability

Among the 339 embryos that were transferred, four in the FCS group and 16 in the CRYO3 group were morphologically damaged (p = 0.01). Nevertheless, during the in vivo evaluation, the implantation rate (21.8%) and the live-foetuses rate (18.8%) of the CRYO3 group were significantly higher than the implantation rate (7.1%, p = 0.0002) and the live-foetuses rate (5.3%, p = 0.0002) of the FCS group. The pregnancy rate was also higher in the CRYO3 group compared to the FCS group (81.3% and 43.8%, respectively, p = 0.066).

## Discussion

This study reports the combination of a physical approach and a biological approach to characterise mammalian embryo slow-freezing solutions. The number of tests we performed for each thermodynamic characterisation was higher than what is normally used for DSC testing. Consequently, we believe that the observed differences in the solutions' physical characteristics are representative of the thermodynamic behaviour of cryopreservation solutions.

During the thermodynamic approach, the variability of the results was higher than the measurement uncertainty of DSC and was observed with the control solution as well, eliminating the explanation that only the macromolecular components contributed to these variations in the cryopreservation solutions. As previously hypothesised, heterogeneity in the cryopreservation solutions could engender variability in the samples for the same solution and consequently cause variable results [29]. To our knowledge, no study has evaluated the biological impact of this variability. This impact is consequently still questionable. Nevertheless it is probably not negligible. On the one hand, the maximal difference observed within one solution between the highest and lowest values of *T*_m_ was about 1.4°C. *T*_m_ is the temperature under which crystallisation can occur. Consequently, a variation in *T*_m_ leads to a variation of the supercooled state at the time of the seeding. Supercooling is one of the most important factors that determine the morphology and surface area of extracellular ice [40], [41]. In the same way, supercooling can precipitate the formation of lethal intracellular ice. Gosden observed that a variation of only 3°C of the supercooled state engenders a difference in ice structure [40]. A difference of 1.4°C of supercooling could thus have similar effects, although attenuated. It seems consequently reasonable to suppose that this difference could have a biological effect. On the other hand, Δ*H* can be used to calculate the maximal heat of ice crystallisation (*Q*_max_): *Q*_max_(%)  =  Δ*H*/Δ*H*_water_ ×100. *Q*_max_ evaluates the mass (g) of ice whose solidification at 0°C would liberate the same amount of heat as that of 100 g of solution on crossing the corresponding peak [42]. Thus, *Q*_max_ is close to the maximal quantity of ice crystallised by percentage solution (w.w^−1^) [43]. The maximal difference observed within a solution between the highest and lowest values of *Q*_max_ was about 4%. Whether this 4% difference in the quantity of ice crystallised in a solution has an impact on the structural damages made to the embryos during freezing is still uncertain. However, this question seems all the more relevant as an aberrant value of Δ*H* was measured with one solution containing FCS.

The three different FCS solutions had similar mean thermodynamic characteristics but different variabilities. Nevertheless, excluding the aberrant value, the differences between the variabilities were much lower than the variabilities within the solutions. Consequently, these differences had probably a lower influence on the biological properties of the FCS solutions than the variability within one FCS solution. The similarity between the thermodynamic properties of the FCS solutions may occur because the cryopreservation solutions contained only 18% FCS, which could limit the impact of the differences in FCS compositions (data provided by suppliers). This hypothesis was reinforced by the small influence of FCS on the mean thermodynamic properties of the cryopreservation solutions. Concerning the differences between the variabilities, two hypotheses can be proposed. Firstly, some FCS compositions could decrease the homogeneity of the cryopreservation solutions and thus increase the results' variability. However, this phenomenon does not explain why the same FCS is not the most stable for *T*_m_, *T*_c_ and Δ*H*. Consequently, we hypothesised secondly that the FCS composition could also cause variations in ice formation or melting. In fact, FCS is a complex solution with many undefined components that could interfere with nucleation processes or ice crystal growth phenomena. The variability in the results could thus be due to the origin of the FCS as well as its industrial processing.

We observed that the *T*_m_ and Δ*H* values for the CRYO3 solution had the same order of magnitude as those obtained with the FCS solutions. The difference between the mean values of Δ*H* was within the DSC's measurement uncertainty, while that between the mean values of *T*_m_ was only slightly higher than the measurement uncertainty of the DSC. Moreover, both differences were lower than the lowest variabilities observed for all of the tested solutions. Similarly, the variability of the CRYO3 solution was similar (*T*_m_) or only slightly higher (Δ*H*) than the lowest variabilities obtained for the FCS solutions. In contrast to FCS, no outlying values were observed in the CRYO3 solution. Thus, we believe that the CRYO3 can replace FCS in rabbit embryo cryopreservation solutions from a physical point of view.

Concerning the biological approach, the embryo survival rates after *in vitro* culture for both the FCS group and the CRYO3 group were similar to those obtained in other studies [44]–[47]. Moreover, no significant differences were observed and the number of morphologically damaged embryos was low and strictly identical between the two groups.

During the *in vivo* evaluation, the implantation and live-foetuses rates obtained with the CRYO3 group were significantly higher than those obtained with the FCS group. The pregnancy rate obtained with the CRYO3 group was also much higher than that obtained with the FCS group but no significant differences were observed. These results are surprising in so far as the embryo survival rates obtained during the two *in vitro* evaluations of viability were similar between the two groups, including the second evaluation. It is difficult to explain this discrepancy between the *in vitro* and *in vivo* survival rates and we have to remain cautious with the two explanations we propose. On the one hand, in animal-derived products, undefined compounds, especially peptides, may be bound to albumin [16]. On the contrary, the synthetic origin of CRYO3 makes the presence of undefined compounds almost impossible. The presence of undefined compounds could induce deleterious effects, which could particularly affect the zona pellucida and could lead to difficulties in the embryo hatching. These effects would consequently not have an influence on the *in vitro* embryo survival rates in contrast to the *in vivo* embryo survival rates. On the other hand, slow-freezing protocols alter the gene expression of rabbit embryos [48]. It is possible that the genetic alterations induced by the FCS-based solutions could lead to more *in vivo* embryo death than those induced by the CRYO3-based solutions. These two phenomena could explain why CRYO3 improved the *in vivo* survival rate of rabbit embryos.

Otherwise, we noticed that more embryos were damaged in the CRYO3 group than in the FCS group. But, in the CRYO3 group, one straw contained eight damaged embryos out of 15 embryos whereas the other 17 straws contained between zero and one damaged embryo. We suspect this could be due to a bad manipulation of this straw. This phenomenon explains for the most part the difference between the numbers of damaged embryos within the two groups. Moreover, this difference in the number of damaged embryos is not correlated with lower *in vivo* viability of the CRYO3 group.

The viability of frozen-thawed embryos after transfer can be very variable. Thus, in the literature, the parturition rate (number of doe giving at least one live-born pup/total recipient does) can range from 59% [49] to 100% [44] and the live-born rate (number of living pups/number of embryos transferred) can range from 19% [45] to 55% [50]. This variability can be due to numerous factors such as the genetic of the embryos, the genetic of the does, the housing conditions, the feeding conditions… In this study, we used the pregnancy rate and the live-foetuses rate that can be compared to the parturition rate and the live-born rate respectively. The pregnancy rate obtained with the CRYO3 group was similar to the parturition rates obtained in other studies [49], [51]. In the same way, the live-foetuses rate was similar to the lowest live-born rates obtained in other studies [45], [51]. This is all the more important as the pregnancy rate and the live-foetuses rate obtained with the control group (FCS group) were lower than the parturition rates and the live-born rates obtained in other studies [44], [45], [49]–[51]. Consequently, we assert that the CRYO3 can replace FCS in rabbit embryo cryopreservation solutions from a biological point of view. The composition of the CRYO3 is confidential because of proprietary interest of the company. However, the presence of a high molecular weight synthetic polymer probably plays the same role as does BSA in FCS and can partially explain these results.

In conclusion, a chemically defined substitute (CRYO3) produced similar or better results when used in embryo cryopreservation solutions compared to solutions containing animal-derived products. The cryopreservation solution containing this substitute had similar thermodynamic and *in vitro* biological properties to those of FCS-containing solutions whereas its *in vivo* biological properties were much better. The CRYO3 had two main advantages. First, its synthetic origin contributes to decrease the sanitary risks during embryo transfer. Second, the CRYO3 solution was more stable than those containing FCS because it showed no outlying data points during thermodynamic testing. We conclude that CRYO3 can be used as a chemically defined substitute in rabbit embryo cryopreservation solutions.

The thermodynamic approach also showed that DSC can be used to study cryopreservation solutions, particularly for evaluating chemically defined substitutes for animal-based products. However, determining how the *T*_m_ and Δ*H* influence the biological properties of the cryopreservation solutions requires additional data. Two follow-up research areas exist. On the one hand, it could be interesting to evaluate the range of thermodynamic properties in which the biological properties remain equivalent, which could be realised by making infinitesimal modifications to CRYO3's composition to slightly modify its physical properties while maintaining the same biological properties. On the other hand, the thermodynamic properties of the cryopreservation solutions could be used to better understand the cryopreservation protocols and consequently to adapt the cryopreservation protocols to the cryopreservation solutions [52].

## Materials and Methods

During the experiment, FCS was used as a reference because it is a common macromolecular source. However, many different FCSs are available from suppliers. Consequently, three FCSs were selected for thermodynamic evaluation (Ref. F2442, Sigma, France, FCS 1; Ref. BWSTS181L/100, VWR, France, FCS 2; and Ref. 10500–056, Invitrogen, France, FCS 3). A cryopreservation solution without any macromolecular component was characterised using DSC and was used as the control. Cryopreservation solutions containing one of the three FCSs were evaluated using DSC to *(i)* evaluate the homogeneity of thermal properties of cryopreservation solutions containing different FCSs, *(ii)* evaluate the influence of FCS on the thermodynamic properties of cryopreservation solutions and *(iii)* select the most thermodynamically stable serum for use in the biological evaluation. A cryopreservation solution containing CRYO3 was also evaluated using DSC to compare its calorimetric properties with those of solutions containing serum.

The biological properties of the CRYO3 or FCS solutions were then evaluated *(i)* by *in vitro* culture and *(ii)* by embryo transfer of frozen/thawed rabbit embryos. Rabbit embryos were chosen because they are good models for mammalian reproduction, early embryology and biomedical research [29], [44], [49], [53].

### Elaboration of cryopreservation solutions

The same buffer and the same cryoprotectant were used for all of the cryopreservation solutions in both the thermodynamic and biological approaches. Consequently, all cryopreservation solutions consisted of Dulbecco's phosphate buffer saline with CaCl_2_ and MgCl_2_ (D-PBS, Ref. 9662, Sigma, France) supplemented with either 10% (v.v^−1^) Dimethyl Sulfoxide (DMSO, Ref. D2650, Sigma, France) plus 18% (v.v^−1^) of any macromolecular component tested (FCS or CRYO3) or 10% DMSO alone (control solution).

All tested cryopreservation solutions were prepared simultaneously to ensure greater homogeneity in their production. A “basic” solution with D-PBS and DMSO was firstly made and was then used to prepare the complete cryopreservation solution containing 18% (v.v^−1^) macromolecular components. For the control solution, 18% (v.v^−1^) of D-PBS was added to the basic solution. All solutions were protected from light before use.

### Thermodynamic evaluation using DSC

The phase transitions of the cryopreservation solutions were characterised using a power compensation DSC (Diamond DSC, Perkin-Elmer, Waltham, Massachusetts, USA) equipped with a liquid nitrogen cooling accessory (Cryofill) and the Pyris software (R9.1 version). The DSC was calibrated for temperature and energy with two standards: the ice melting of reverse-osmosis purified water (0.00°C; Δ*H*_water_  = 333.00 J.g^−1^) and the crystallographic transition of cyclohexane in its solid state (−87.06°C; 79.58 J.g^−1^). The validity of the calibration was verified regularly using tests on reverse-osmosis purified water. Experiments were conducted using standard hermetically sealed aluminium pans (Ref. 0219–0062, Perkin-Elmer, Waltham, Massachusetts, USA) designed for volatile samples.

After the calibration, additional tests were performed on reverse-osmosis purified water and cyclohexane to verify the accuracy of the DSC under our experimental conditions. The maximal measurement uncertainty obtained with these pure standards was 0.11°C (temperature) or 1.14% (energy), which was consistent with the information provided in the technical specifications of the DSC (±0.11°C for temperature and ±1.10% for energy).

During the experiment, the cryopreservation solutions were stored at −20°C in small glass vials with a Teflon cap and were protected from light before use. The glass vials were previously cleaned and sterilised to remove any impurities that could interfere with the thermodynamic measures. One glass vial containing a cryopreservation solution was used for only one test. Nine replicate measurements were taken for each solution. During each test, one glass vial was placed at room temperature for approximately 20 min to thaw the cryopreservation solution. The solution was then loaded in aluminium pans (20 µL) using a micropipette to limit the variations in weight between the samples. The pans were previously cleaned following the standard procedure provided by Perkin-Elmer. The aluminium pans were first weighed without cryopreservation solution on a precision weighing balance (AE240, Mettler, Suisse) and were then weighed after the loading of the cryopreservation solution in order to measure the sample mass. The weights were determined with a negligible error of less than 0.02 mg. To ensure the sealed pans' insulation, sealed pan weights were measured again at the end of the experiments and were compared with the weight obtained before the DSC measurements. In these conditions, the average mass of sample was 5.47 mg±0.57 (mean ± standard deviation, *n* = 45).

Two cycles of cooling and warming between 10°C and −150°C were applied to each sample to determine the *T*_m_ and Δ*H* of the cryopreservation solutions. These characteristics were chosen because they allow a good characterisation of solutions used in slow-freezing protocols. *T*_m_ is the temperature under which crystallisation can occur, and Δ*H* allows the quantification of crystallised ice in the solution. The intervals between successive warmings and coolings were short because the water in aqueous solutions has a tendency to evaporate and condense as very tiny droplets at the top of the sample holders.

First, a rapid cooling (100°C.min^−1^) was followed by a slow warming (2.5°C.min^−1^) to measure *T*_m_. Despite our precautions to avoid water evaporation, a small parasitic peak was systematically observed above the main one during this first warming, as previously described [43], which increased on successive warmings (unpublished results). This peak was probably due to the equilibration time of the sample required prior to running experiments. Because its area was much smaller than that of the main peak, this small parasitic peak was not taken into account for measuring the *T*_m_. This temperature was defined at the top of the main melting peak.

Second, a slow cooling (2.5°C.min^−1^) was followed by a rapid warming (20°C.min^−1^) to determine Δ*H*. Δ*H* was measured by evaluating the area encompassed between the peak of crystallisation and the baseline.

A third thermodynamic characteristic was also determined: the *T*_c_ measured during slow cooling. However, in contrast to *T*_m_ and Δ*H*, which depend mainly on the sample composition, *T*_c_ is strongly dependent on the experimental conditions because it corresponds to the end of the supercooled state. In fact, in these bulk and large cryoprotective solution samples, essentially heterogeneous nucleation happens due to impurities in the liquid or defaults in the contact surface of the aluminium pan [54]. The larger the impurities or defaults are, the higher the temperature for nucleation becomes, and the higher the value of *T*_c_ is. Consequently, *T*_c_ may be variable and was only used to choose the most thermodynamically stable FCS for the biological evaluation. *T*_c_ was measured at the onset of the crystallisation peak during slow cooling at 2.5°C.min^−1^.

### Biological evaluation using rabbit embryo freezing

#### Ethic statement

This study was carried out in strict accordance with the European Guideline 86/609/EC of 1986 for the care and use of animals for scientific purposes. The protocol was approved by the Committee on the Ethics of Animal Experiments of VETAGRO SUP – Veterinary campus of Lyon (Permit Number: 05/26).

#### Slow cryopreservation and thawing

Hormonal treatment of the rabbit does and the ensuing embryo recovery were performed using the protocol described by Salvetti et al. [44]. Briefly, fertile rabbit does (New Zealand cross breed, SARL HYCOLE, Marcoing, France) were superovulated using five subcutaneous injections of pFSH (Stimufol®, Reprobiol, France). The recovered embryos from each doe were divided evenly between two groups: (i) the FCS group, which was frozen in a cryopreservation solution containing 18% FCS 1, and (ii) the CRYO3 group, which was frozen in a cryopreservation solution containing 18% CRYO3.

A total of 555 embryos (277 in the FCS group and 278 in the CRYO3 group) were frozen using the slow-freezing protocol described by Joly et al. [49]. Briefly, the embryos were firstly equilibrated at room temperature for five min in three successive baths containing D-PBS, the chosen macromolecular component and increasing concentrations of DMSO: 0.5 M, 1 M and 1.5 M. At the end of equilibration, 5–25 embryos were loaded into 0.25 mL French sterile straws (IMV, France), which were sealed by a sterile plug.

The straws were then placed directly in a programmable freezer (Cryocell 1200, IMV, France) previously equilibrated at −7°C. After five min of equilibration, a liquid nitrogen pre-cooled swab was used to induce the seeding. After a second equilibration period of ten min, the embryos were cooled to −30°C at a freezing rate of 0.5°C/min. Finally, the straws were plunged directly into liquid nitrogen where they were stored for one week.

For thawing, the straws were placed in ambient air for 10–15 sec until the ice crystals disappeared before plunging them into a water bath at 20°C for one min. The contents were then emptied into a petri dish in which the embryos were collected. Once collected, the embryos were placed in three successive baths at room temperature for five min to remove the DMSO and to rehydrate the embryos. The first two baths consisted of IMV holding medium (IMV, France) supplemented with decreasing concentrations of DMSO (1.0 M and 0.5 M), while the final bath contained only IMV holding medium.

#### First assessment of viability: in vitro culture of frozen/thawed embryos

Two in vitro evaluations were performed. In fact, the first in vitro evaluation and the in vivo evaluation were separated in time. The second evaluation was consequently performed at the same time as the in vivo experiment to evaluate the in vitro survival rates obtained with the embryos used for embryo transfers. Seventy six embryos and 32 embryos of each group were cultured during the first and the second in vitro evaluation respectively.

After thawing, embryos of each group were cultured in KSOM (EmbryoMax^®^ KSOM Embryo Culture, Millipore, France) at 38.5°C under 5% CO_2_ in air and a saturated humidity atmosphere. After 48 h of *in vitro* culture, the embryo survival rate was evaluated by assessing the ability of thawed embryos to develop into the blastocyst or expanded blastocyst stage.

#### Second assessment of viability: embryo transfer of frozen/thawed rabbit embryos

After thawing, 169 embryos of the FCS group and 170 embryos of the CRYO3 group were transferred on 32 synchronised recipients does (New Zealand cross breed, SARL HYCOLE, Marcoing, France).

Embryo transfer was performed using the protocol described by Salvetti et al. [44]. Briefly, an intramuscular injection of Buserelin acetate (Receptal^®^, Intervet, France) induced ovulation 50–60 h before transfer. After anesthesia, an average of 10 embryos was transferred into the uterine horns to each recipient doe via a midventral laparotomy.

Fifteen days after embryo transfers, the *in vivo* development viability of thawed embryos was evaluated using the pregnancy rate (number of doe containing at least one foetus/total recipient does), the implantation rate (number of implantation sites/total embryos transferred into recipient) and the live-foetuses rate of the transferred embryos (number of living foetuses/total embryos transferred into recipient).

### Statistical analysis

Statistical analyses were performed using the R software [55].

The normality of the thermodynamic results was verified using the Shapiro-Wilk normality test. The calorimetric values obtained with FCS solutions did not exhibit Gaussian distributions. The comparison of the thermodynamic properties of solutions was performed using descriptive statistics of two criteria: their mean values and the variability of their values.

The embryo survival rates after *in vitro* culture and the *in vivo* development viability between the FCS group and the CRYO3 group were compared using Fisher's exact test. Differences with p<0.05 were considered as significant.
